# Use of the Arthrex ProStop Subtalar Arthroeresis Screw in the Management of Symptomatic Paediatric Flexible Flatfoot

**DOI:** 10.7759/cureus.72151

**Published:** 2024-10-22

**Authors:** Matthew Langstroth, Ghazal Hodhody, Qaisar Choudry

**Affiliations:** 1 Department of Trauma and Orthopaedics, East Lancashire Hospitals National Health Service (NHS) Trust, Blackburn, GBR

**Keywords:** flexible flatfoot, paediatric foot and ankle, paediatric foot deformities, paediatric orthopedics, subtalar arthroereisis

## Abstract

Introduction: Paediatric flexible flatfoot (PFFF) is a common, potentially debilitating condition affecting a significant proportion of active children. Despite its prevalence, there is a lack of consensus on optimal operative management in symptomatic children. We report a unique case series of six feet treated with the Arthrex ProStop Subtalar Arthroeresis Screw (Arthrex, UK) in the North West of England.

Methods: Following a comprehensive review of available literature, we performed a retrospective study of six feet (four patients, aged 11-15 years, including one female and one male with bilateral disease) managed with the Arthrex ProStop Subtalar Arthroeresis Screw from 2019 to 2023. Radiographic and clinical findings pre- and post-operatively were compared with a two-year follow-up.

Results: Post-operative radiographs demonstrated an increase in calcaneal pitch, an average of a 17.6° improvement in Meary's angles and a reduction in talonavicular joint uncoverage. Clinical improvement was seen in both stance and heel valgus. The intervention resulted in improved patient satisfaction in all. In this series, there were no infections. However, one implant was removed at 14 months due to persistent pain. All remaining screws were removed at two years due to a lack of research into the long-term outcomes of the device in paediatric patients.

Conclusions: This study highlights that subtalar arthroereisis is an effective surgical management option for treating PFFF in adolescents, with limited available literature. Further, large-scale comparative studies with long-term follow-up are required to delineate the true benefit of this procedure.

## Introduction

Paediatric flexible flatfoot (PFFF) is a common yet challenging presentation affecting a large proportion of the paediatric population, with some studies reporting a prevalence of between 20% and 70% [1,2]. However, prevalence values may well be underestimated due to asymptomatic cases not presenting to healthcare professionals [3]. Considered a physiological deformity until the age of 10, PFFF is characterised by flattening of the medial arch, valgus deformity of the hindfoot and abduction of the forefoot during weight-bearing, with restoration of the physiological arch when non-weight-bearing and upon dorsiflexion of the great toe, distinguishing it from rigid pes planovalgus [3,4]. PFFF can occur as a primary disorder or secondary to systemic diseases that result in ligamentous laxity and hyper-mobility of the subtalar joint, including Ehlers-Danlos and Marfan syndrome [4]. Although a well-recognised and prevalent condition, there is a lack of universally accepted criteria for diagnosing PFFF, resulting in the reported prevalence being highly variable in literature [2,3].

The physiological mechanisms for maintaining the normal foot arch and, subsequently, the pathogenesis of PFFF have been the subject of academic debate for some time, with multiple proposed theories [2-4]. There is now some consensus that the pathology is primarily due to excessive ligamentous laxity within the foot, resulting in the deformity previously described [3]. There remains much debate as to what is considered pathological; hence, the management of PFFF remains highly controversial [3].

The diagnosis of PFFF is made on clinical examination of a child presenting with the characteristic deformity, with the restoration of the longitudinal arch on the removal of weight-bearing and dorsiflexion of the great toe [3,4]. PFFF can exist in isolation or may be associated with various other deformities, including Achilles tendon contractures, which, according to Mosca's study in 2010, can permit further sub-classification and subsequently aid treatment decisions for PFFF [3]. Although a clinical diagnosis, plain film radiographs are commonly used in further assessment of cases of PFFF, with multiple established radiographic measurements utilised in assessing the severity of the deformity, including Meary's and Kite's angles, as well as talonavicular uncoverage and calcaneal pitch [2,3,5]. Weight-bearing radiographs have been deemed more adequate for detecting and quantifying flatfoot and in making treatment decisions [6].

The optimal management of PFFF remains unclear, with a multitude of options described in the literature [3-5]. PFFF can be managed expectantly where asymptomatic; however, when symptomatic, it can initially be managed conservatively with a range of options, including orthoses. Surgery is often reserved for persistently symptomatic cases despite conservative management [5]. The primary aim of management should be to enable the child to be pain-free and return to function, including sporting activities.

Although a highly prevalent condition, only a small proportion of children with PFFF develop disabling symptoms, such as chronic pain [3], and a further small percentage of this group goes on to require operative management. The lack of a generalised consensus exists for both when to consider operative management and the best practice for operative management, with a range of options available [3-5,7,8]. Earlier literature can be interpreted to indicate that there is no role for operative management of PFFF in the absence of an associated short Achilles tendon [3]. Previously, various operative techniques have been performed, ranging from soft tissue tensioning techniques to osteotomies [3,9]. Arthroereisis, which involves the insertion of an implant into the sinus tarsi and subsequent stabilisation of the subtalar joint and surrounding physiologic anatomical remodelling [7], is now a more commonly utilised operative procedure for PFFF. Considering this option alone, multiple devices and techniques are cited in the literature, including the use of both bioabsorbable and non-bioabsorbable implants [8].

The Arthrex ProStop device (Arthrex, UK) is one arthroereisis implant option. It is a non-bioabsorbable, soft-threaded, conically shaped arthroereisis screw that can be inserted via a minimally invasive technique to open the sinus tarsi, reduce hyper-pronation at the sub-tarsal joint and subsequently correct the PFFF deformity during weight-bearing [10]. The implant then results in physiological remodelling, such that on removal, there is continued correction of the flatfoot deformity [11].

There remains a scarcity of literature regarding the use and subsequent outcomes of arthroereisis in treating PFFF in the United Kingdom. We report a case series of six feet (four patients) treated with the Arthrex ProStop device in a District General Hospital in the North West of England between 2019 and 2022.

## Materials and methods

**Figure 1:**
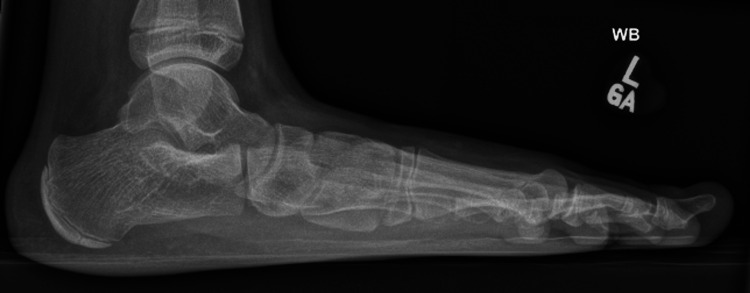
A pre-operative weight-bearing lateral radiograph of the left foot demonstrating a low calcaneal pitch and negative Meary's angle

**Figure 2:**
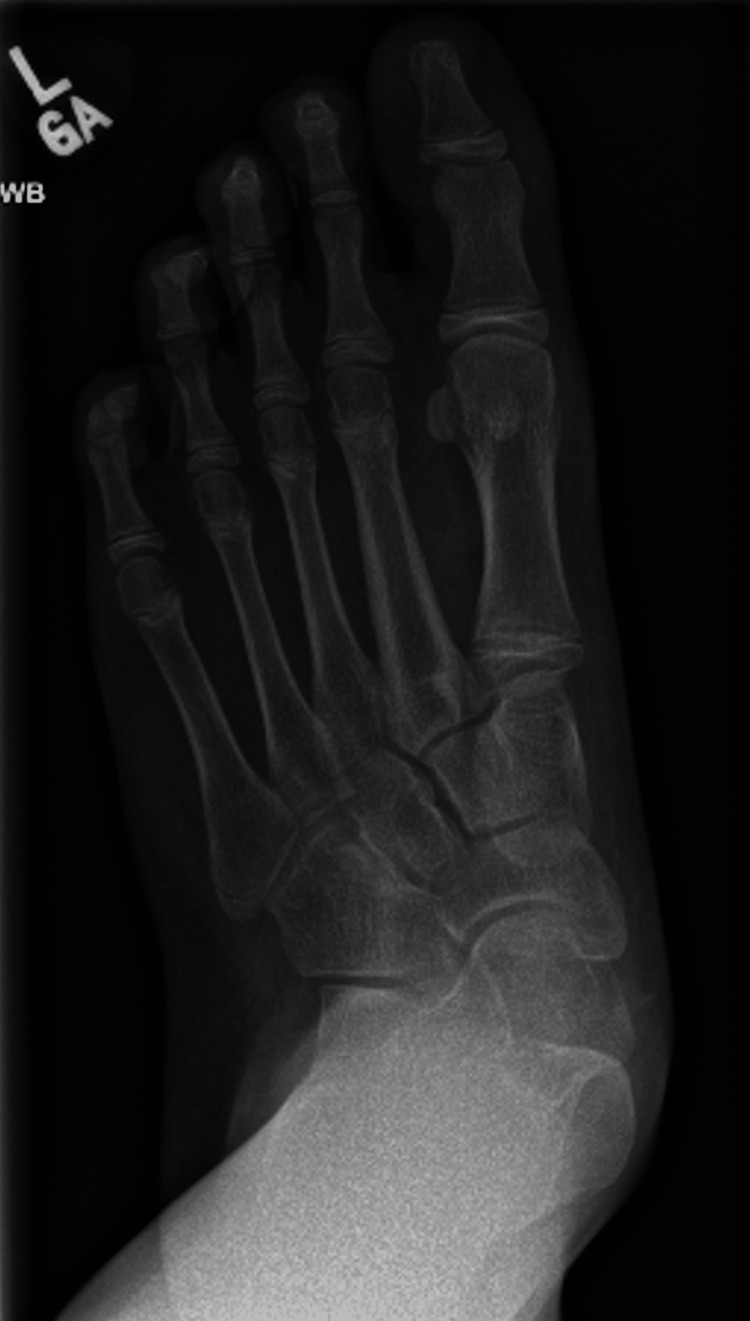
A pre-operative weight-bearing DP radiograph of the left foot, demonstrating increased talonavicular uncoverage and an increased Kite's angle DP: dorsoplantar

**Figure 3:**
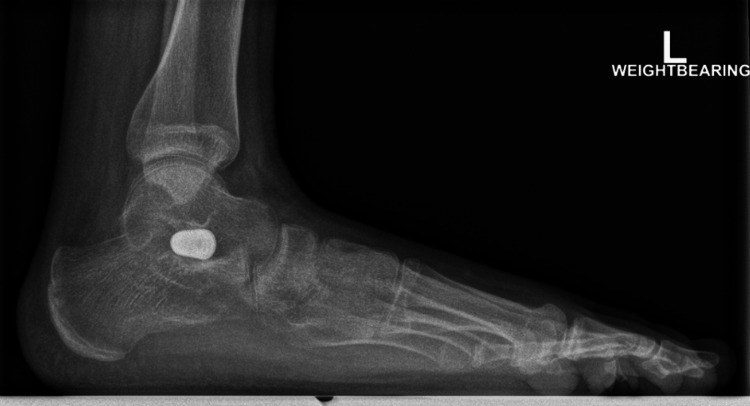
A post-operative weight-bearing lateral radiograph of the left foot, demonstrating improvements in the calcaneal pitch and Meary's angle

**Figure 4:**
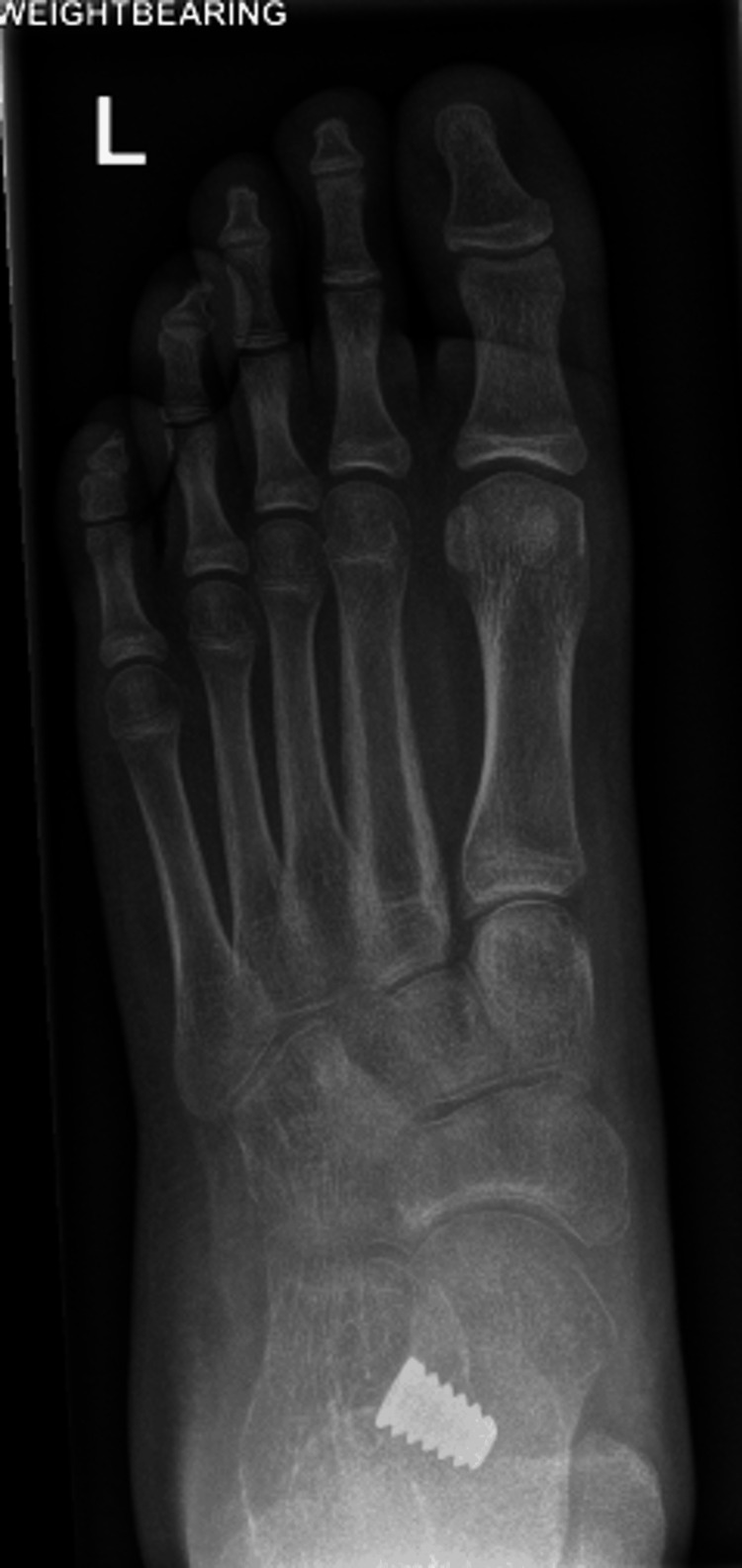
A post-operative weight-bearing DP radiograph of the left foot, demonstrating improvements in talonavicular uncoverage and Kite's angle DP: dorsoplantar

We thoroughly reviewed all currently available literature relating to operative management of PFFF using arthroereisis, drawing on Medline, Cumulated Index in Nursing and Allied Health Literature, Embase, Emcare Allied and Complementary Medicine Database and the Cochrane Library. This comprehensive search revealed only 73 relevant articles, which we then individually analysed. The case series we describe is a single-centre, single-surgeon study, retrospectively analysing six cases (six feet, four patients), aged between 11 and 15 years, operatively managed with arthroereisis, using the Arthrex ProStop device (Figures 1-4), at a single centre in the North West of England between 2019 and 2022. All cases consented to inclusion in and publication of this research.

Each case underwent pre-operative clinical and radiographic assessment, with Silfverskiold testing and gait assessment in the clinic, followed by radiographic assessment of Kite's and Meary's angles, talonavicular uncoverage and calcaneal pitch. All six cases had pre- and post-operative X-ray assessments. Additional pre-operative imaging was sought in three patients: two underwent magnetic resonance imaging (MRI) scanning and one underwent computerised tomography (CT) assessment. It was planned for all patients to undergo routine operative removal of the screw two years post-insertion due to the lack of literature surrounding the long-term use of the device in paediatric patients. This was the decision of the operating surgeon. The average duration of follow-up to discharge from the time of the primary procedure was 30.5 months (19-46 months).

## Results

Of the six cases (four patients) described in this series, two had bilateral disease, with a total of four left feet and two right feet and a mean age of 13 years. Before operative intervention, three of the six cases were managed with orthoses in the form of insoles, with the remainder proceeding directly to operative management. All patients were operatively managed using the Arthrex ProStop system. As previously noted, all underwent pre- and post-operative plain radiograph assessments, with three cases undergoing further detailed assessment with MRI (two out of six) and CT (one patient) scanning. In this series, it was planned for all cases to undergo elective screw removal at two years post-insertion due to a lack of evidence regarding long-term outcomes for paediatric patients managed with this device. This interval was an individual surgeon's choice, resulting from a lack of research into the long-term outcomes of retained implants. At present, there is no clear recommendation of a specific timeline for the removal of implants from Arthrex [10].

It is worth noting that of the six feet operatively managed, one foot had previously undergone a distal tibial derotation osteotomy and fibula osteotomy 22 months before their arthroereisis. At the time of their arthroereisis, they underwent simultaneous Achilles tendon lengthening.

Following operative intervention, clinical improvements in stance and heel valgus were noted in all patients, along with improvements in patient satisfaction. At the two-year follow-up, all patients and families were satisfied with the outcome. With respect to radiographic measurements (Table 1), there was a significant post-operative improvement, with a mean reduction in Kite's angles of 13° (2.8-22) and a 17.6° mean increase in Meary's angles (5.1-34), as well as improvements in talonavicular uncoverage and calcaneal pitch.

**Table 1:** Comparison of the mean and range of key radiographic values from pre- and post-operative plain film imaging

Radiographic measurement	Pre-operative mean	Pre-operative range	Post-operative mean
Kite's angle	35.4°	30.2°-39.5°	22.5°
Meary's angle	-23.9°	-37.5° to -9.2°	-6.3°
Talonavicular uncoverage	51.3%	41.5%-59.6%	31.4%
Calcaneal pitch	11.9°	9.5°-16.2°	14.2°

During post-operative follow-up, two (33%) complications were identified. One patient developed peroneal spasticity following insertion of the screw, which was subsequently removed at 14 months and not re-inserted. The device prematurely backed out in the second case, resulting in a re-insertion procedure. In the latter, the device was subsequently well tolerated until routine removal at two years, as planned for all in this study.

## Discussion

There remains controversy around the topic of PFFF. There is ambiguity around the nature of PFFF and the determination of what is classified as pathological or considered a normal physiological variant [3,9]. Indeed, a patient may present with a flatfoot that may clinically be considered pathological but lack any associated symptoms, as these patients commonly present due to parental concerns regarding the physical appearance of the foot [3]. In 2014, Bouchard and Mosca noted that flexible flatfoot in the absence of pain is a normal physiological variant and, therefore, should be conservatively managed [9]. The controversy continues even when subsequently accepting that management should only be commenced in symptomatic children. Not only is there scanty evidence surrounding the justification of using orthoses in treating PFFF, but some researchers have demonstrated that they can have significant negative psychological impacts on the wearer, including reducing the child's self-esteem [12]. It is also well-documented that orthoses do not result in any significant, permanent physiological remodelling of the foot [3,13].

Operative management of patients with PFFF is still frequently practiced. There remains debate about the optimal choice of surgical treatment, with multiple techniques previously being utilised, including multi-joint arthrodesis, soft tissue tensioning techniques, osteotomies and subtalar arthroereisis [3-5,7-9,11]. Subtalar arthroereisis itself remains controversial, with multiple potential implant options and techniques and well-documented complications, including persisting pain [9].

Our case series supports the limited available evidence that subtalar arthroereisis via minimally invasive approach and screw insertion is a well-tolerated, safe and suitable management option for the treatment of symptomatic PFFF, including when used in bilateral disease [8,11,14-16]. Furthermore, it highlights that this technique results in radiographic and physiological improvements in the PFFF deformity.

With respect to the limitations of this study, we first acknowledge the small sample size and the single-centre, single-surgeon nature of the study. However, despite the reported prevalence of PFFF, as we previously described, only a small proportion of cases are symptomatic, and a subset of this cohort then goes on to have operative management. Subsequently, there is currently limited available evidence for optimal surgical management of these cases. Hence, this valuable research contributes to the available data in favour of the use of arthroereisis. We also acknowledge the lack of long-term guidance regarding the optimal timing of implant removal, with subsequent surgeon choice of removal of all implants at two years, arising from the lack of evidence surrounding the long-term safety and outcomes for retaining the device. There is a clear deficiency in the current literature and scope for further study to determine the optimal timing for implant removal and assess the maintenance of the anatomical changes to the foot secondary to the device. Finally, this is a single-surgeon, single-centre study assessing children managed with a single operative device and technique. Whilst considering this, there is minimal variation in underlying operative principles between the subtalar implants used.

Within our cohort, we note the occurrence of complications in two (33%) of the assessed feet. The complications consisted of premature screw back-out and secondary peroneal spasticity. However, in both cases, despite these complications, at the two-year follow-up, both remained happy with their outcomes, with clinical and radiographic improvements. Complications of arthroereisis are well documented in the literature, with reported rates varying between 3.5% and 40% [3,8], with premature device back-out and device-induced peroneal spasticity, as noted in our study, being well recognised as potential complications of treatment [3]. However, alternative operative techniques for the management of PFFF are not without their complications, with techniques such as osteotomies requiring a longer, more invasive procedure and lacking the option of quick and easy reversal when compared to the removal of an arthroereisis screw [17].

## Conclusions

Subtalar arthroereisis using the Arthrex ProStop device is a safe and effective method for managing symptomatic PFFF, which can be performed via a minimally invasive approach, with clinical and radiographic improvements seen in all within this series. There is a clear lack of literature regarding long term use of arthroereisis screws and the safety of retaining these implants. This study highlights the need for further, long-term, large-scale comparative research in several key areas surrounding the use of subtalar arthroereisis in the treatment of PFFF. Most importantly, this would enable accurate determination of the efficacy of this technique, as well as providing greater clarity on the true complication rate. Finally, this would enable determination of optimal timings for removal of non-bioabsorbable implants and the subsequent permanence of the anatomical remodelling of the foot resulting from the use of subtalar arthroereisis.
